# Oral human Papillomavirus DNA detection in HIV-positive men: prevalence, predictors, and co-occurrence at anal site

**DOI:** 10.1186/s12879-017-2937-0

**Published:** 2018-01-08

**Authors:** Alessandra Vergori, Anna Rosa Garbuglia, Pierluca Piselli, Franca Del Nonno, Catia Sias, Federico Lupi, Daniele Lapa, Andrea Baiocchini, Claudia Cimaglia, Marco Gentile, Andrea Antinori, Maria Capobianchi, Adriana Ammassari

**Affiliations:** 1 HIV/AIDS Unit, INMI “L.Spallanzani”, Rome, Italy; 2 Laboratory of Virology, INMI “L.Spallanzani”, Rome, Italy; 3 Clinical Epidemiology Unit, INMI “L.Spallanzani”, Rome, Italy; 4 Laboratory of Pathology, INMI “L.Spallanzani”, Rome, Italy; 5 National Institute for Infectious Diseases, IRCCS L.Spallanzani, Via Portuense, 292, 00149 Rome, Italy

**Keywords:** HPV, HIV, Oral cavity, Anal site, Vaccine-targeted infections

## Abstract

**Background:**

HIV-positive patients carry an increased risk of HPV infection and associated cancers. Therefore, prevalence and patterns of HPV infection at different anatomical sites, as well as theoretical protection of nonavalent vaccine should be investigated. Aim was to describe prevalence and predictors of oral HPV detection in HIV-positive men, with attention to nonavalent vaccine-targeted HPV types. Further, co-occurrence of HPV DNA at oral cavity and at anal site was assessed.

**Methods:**

This cross-sectional, clinic-based study included 305 HIV-positive males (85.9% MSM; median age 44.7 years; IQR: 37.4–51.0), consecutively observed within an anal cancer screening program, after written informed consent. Indication for anal screening was given by the HIV physician during routine clinic visit. Paired oral rinse and anal samples were processed for the all HPV genotypes with QIASYMPHONY and a PCR with MY09/MY11 primers for the L1 region.

**Results:**

At the oral cavity, HPV DNA was detected in 64 patients (20.9%), and in 28.1% of these cases multiple HPV infections were found. Prevalence of oral HPV was significantly lower than that observed at the anal site (*p* < 0.001), where HPV DNA was found in 199 cases (85.2%). Oral HPV tended to be more frequent in patients with detectable anal HPV than in those without (*p* = 0.08). Out of 265 HPV DNA-positive men regardless anatomic site, 59 cases (19.3%) had detectable HPV at both sites, and 51 of these showed completely different HPV types. At least one nonavalent vaccine-targeted HPV type was found in 17/64 (26.6%) of patients with oral and 199/260 (76.5%) with anal infection. At multivariable analysis, factors associated with positive oral HPV were: CD4 cells <200/μL (versus CD4 cells >200/μL, *p* = 0.005) and >5 sexual partners in the previous 12 months (versus 0–1 partner, *p* = 0.008).

**Conclusions:**

In this study on Italian HIV-positive men (predominantly MSM), oral HPV DNA was detected in approximately one fifth of tested subjects, but prevalence was significantly lower than that observed at anal site. Low CD4 cell count and increasing number of recent sexual partners significantly increased the odds of positive oral HPV. The absence of co-occurrence at the two anatomical sites may suggest different routes or timing of infection.

## Background

In the current era of combination antiretroviral therapy (cART), tumours have become the leading cause of mortality in HIV-positive persons [1, 2]. Among non-AIDS cancers, HPV-associated tumours recently gained great attention because of multi-site distribution (genital tract, anus, head and neck), availability of screening procedures, and need of vaccine allocation strategies. HIV-positive persons experience a 3-fold higher standardized incidence of head and neck squamous cell carcinoma (HNSCC) than general population [3], and about 50% of oro-pharyngeal squamous cell carcinoma (OSCC) are HPV-related tumours, as found in a large retrospective study that investigated the prevalence of HPV infection in cancer tissues from more than 1600 patients diagnosed in the UK during the last decade [4]. Notably, HIV-positive persons were 2.1 times more likely to harbor HPV in the oral cavity in comparison to HIV-negative subjects [5]. Screening studies conducted in the HIV-infected population demonstrated an overall prevalence of HPV DNA in the oral cavity was between 20 and 45%, with the finding of the oncogenic type HPV16 between 12 and 26% [6–9].

In a meta-analysis of 26 publications focusing on men having sex with men (MSM), pooled prevalence of any HPV at the oral cavity was 17.1% (95% CI 7.3–26.8%) in HIV-negative and 28.9% (95% CI 19.1–38.7%) in HIV-positive MSM [9]. Overall, factors predictive of oral HPV infection were: HIV infection, severe immunodepression and number of sexual partners [9, 10].

Concerning multi-site HPV infections, particularly MSM were at high risk of HPV co-occurrence at multiple anatomic sites, as shown in a study that found 59.1% MSM with multi-site HPV occurrence (34.1% and 22.7% at two and three sites respectively) [11]. Yet, detection of the same HPV types at the oral cavity and the anus can be as low as 5% [7, 9, 12–14]. Yet, evaluation of HPV DNA co-occurrence at diverse anatomic sites should be evaluated in different geographical and clinical settings, because it may have impact on the clinical management of HIV-infected persons with regards to HPV detection and cancer screening strategies.

Vaccines to prevent HPV infection have been developed and are approved in some countries for immunization programs in adolescent females, as well as in boys and young men [15, 16]. Currently, the U.S. Food and Drug Administration has approved the nonavalent vaccine (Gardasil 9 ®, Merck & Co., Inc., Whitehouse Station, NJ) targeting HPV types 6, 11, 16, 18, 31, 33, 45, 52, 58 for prevention of cervical, vulvar, vaginal and anal cancers and ano-genital warts [17–19]. Studies found the oncogenic type HPV16 in the oral cavity among 12 to 26% of cases [6–9], and prevalence of HPV types covered by the nonavalent vaccine in a study on 90 MSM was 77.8% in HIV-positive subjects, with HPV 58 and 16 mostly detected [11]. Since HPV vaccines have never been investigated for the prevention of OSCC, availability of more knowledge regarding the prevalence of nonavalent vaccine-targeted HPV types in HIV-infected patients with detectable HPV at the oral cavity would add some relevant information on the proportion of patients that in theory would have been protected if the vaccine had been administered before exposure.

In Italy at present not much is known about prevalence and predictors of oral HPV DNA detection in HIV-infected patients, and literature about co-occurrence of HPV infection in oral cavity and at anal site is scanty. For this reason, only few HIV/AIDS centres investigate presence of HPV in oral rinses and offer oral cancer screening to their patients. The aim of our study was to describe prevalence and predictors of oral HPV DNA detection in HIV-positive men attending an anal cancer screening program, with attention to vaccine-targeted HPV types. Further, we investigated co-occurrence of HPV DNA at the oral cavity and at the anal site.

## Methods

### Study design and population

This cross-sectional, clinic-based study was designed and conducted at the National Institute for Infectious for Disease, Rome, Italy, according to the Helsinki declaration and approved by the Institutional Review Board (n.42–18/6/2013).

All HIV-positive men, aged 18 years or older, consecutively observed within an anal cancer screening program between February 2015 and June 2016 provided written informed consent. The indication for anal screening was given based on current European AIDS Clinical Society (EACS) guidelines [20] by the HIV physician during routine clinic visit. Patients with history of anal warts or of pre-cancerous lesions were not excluded.

A questionnaire on health behaviours was created ad hoc and interviewer-administered. Questions focused on the following information: demographics, tobacco and alcohol use (more than 3 units per day), previous history of anal/genital condylomatosis, number of sexual partners in lifetime and in the previous 12 months, oral and anal sexual intercourse in lifetime and in the previous 12 months.

Clinical charts were used to abstract presence and type of current cART, as well as viroimmunological parameters (CD4 cell count and HIV RNA needed to be determined within 3 months of samples collection).

### HPV DNA testing

At the same time of anal cancer screening, participants provided a sample of oral rinse obtained by swishing and gargling in the oral cavity 10–15 ml of bottle mineral water (Fonte Tullia) for 20–30 s and spitting into a sterile specimen cup. One aliquot (5–7 ml) was sent to the Laboratory of Pathology for cytological evaluation, while the second was centrifuged for 15 min at 3000 g. Cell pellet was washed with sterile phosphate buffered saline and re-suspended in RPMI medium. Nucleic acids were extracted by automated QIASYMPHONY purification system (QIAGEN, Hilden, Germany). QuantiFast Pathogen Internal Control (QIAGEN, Hilden, Germany), was used to check presence of inhibiting factors. Furthermore, to ensure specimen adequacy, i.e. the presence of DNA, the samples were tested by amplification of a 110 bp fragment of the human β-globin gene using PCO3/PCO4 primers [21] followed by analysis in 2% agarose gel. Samples positive for β-globin were considered evaluable.

For HPV DNA testing, a PCR for the L1 region was used. Products were analysed by electrophoresis in 1.7% agarose gel and stained with ethidium bromide, as previously described [22]. HPV-positive samples by MY09/11 PCR were typed using Genomica CLART assay. Samples, that could not be typed with the CLART assay, were directly sequenced using a Big Dye Terminator Cycle Sequencing Kit (Applied Biosystems, Ca). Sequence alignments were obtained using returned results from GeneBank’s on-line BLAST server (http//https://blast.ncbi.nlm.nih.gov/Blast.cgi, nih.gov/BLAST) [23]. A similarity above 90% was used to identify all known HPV genotypes.

Anal swabs were collected in a standardized manner with a flocked swab according to the manufacturer’s instructions (Eswab, Copan Italia Spa, Brescia, Italy) [24]. The swab was then placed in a sterile tube containing 1 ml of phosphate buffer saline and processed as described above.

Subjects with positive HPV DNA were classified in having a high-risk (HR) HPV, if at least one HR HPV-type was found, or otherwise classified having a low-risk (LR) HPV, if harbouring only low-risk HPV-types. Carcinogenic risk classification was done according to IARC monograph (2007) [25]. Detection was classified in single or multiple HPV if only one or more than one types were characterized. Number of HPV types targeted by nonavalent HPV vaccine were counted in oral and anal samples.

All assays were performed by trained laboratory technicians blinded to clinical information of the patient and to the assay results.

### Cytological examination of oral rinses

Fixed cytological smears were stained according to the Papanicolau method [26] and adequacy evaluated based on the cellularity, as well as on the presence of acceptable fixation and staining.

Two pathologists, blinded to clinical and HPV information, independently evaluated cytological specimens. A cytological assessment of the quality of the obtained cells from each oral sample was undertaken, using standard parameters that include quality of preparation, cellularity, and types of cells present, in addition, data on micro-organisms, leucocytes/inflammatory cells and artefacts were also obtained. Cytological findings were reported using the following categories of the Bethesda 2001 guideline [27] modified as appropriate for the site: NILM (negative for intraepithelial lesion or malignancy), ASC-US (atypical squamous cells of undetermined significance), L-SIL (low grade squamous intraepithelial lesion), or H-SIL (high grade squamous intraepithelial lesion).

### Statistical analysis

Descriptive analysis was conducted to characterize the subjects included in the study. Median values and interquartile ranges (IQR) were used to describe numerical variables, while counts and percentages were employed for qualitative variables. The number of patients with at least one nonavalent vaccine-targeted HPV type over the total number of subjects found to be HPV DNA-positive at each anatomical site were employed to calculate the proportion of vaccine-targeted infections separately for each anatomical site. The association between variables was assessed using chi-squared test or Fisher’s exact test, as appropriate. Two-tailed *p*-values were calculated and a value <0.05 was considered statistically significant. A univariate logistic regression was used to analyse the strength of association between positive oral HPV detection and other variables, calculating odds ratios (OR) and their 95% confidence intervals (CI). A multivariable regression analysis, adjusting for variables associated with the outcome at univariate analysis with a *p* < 0.10 and forcing age, was carried out. Data management and analysis were performed using SPSS version 23 [28].

## Results

### Study population

The study population included 305 HIV-infected males and general characteristics are shown in Table 1. Briefly, patients were predominantly Caucasians (92.8%), with a median age of 44.7 years (IQR: 37.4–51.0). Overall, 262 (85.9%) were MSM. The remaining patients reported HIV acquisition by heterosexual intercourse in 27 cases (8.9%) and by intravenous drug use in 4 cases (1.3%). Mother-to-child transmission and blood transfusion were registered in 1 case each. In the remaining 10 subjects (3.3%) HIV transmission modality remained unknown. At anal cancer screening, 289 (94.8%) patients were on cART since a median of 4.4 years (IQR: 1.9–9.3). Viroimmunological exams showed HIV RNA below 40 copies/ml in 268 patients (87.9%) and a median CD4 cell count of 699/μL (IQR: 529–878). Sixty-two patients (20.3%) had a CD4 cell count <500 cells/μL and 8 < 200 cells/μL. Self-reported previous or current anal/genital condylomatosis was recorded in 112 (36.7%) men.

**Table 1:** Description of the study population (*n* = 305)

Demographics	
Age, median years (IQR)	44.7 (37.4–51.0)
Caucasian ethnicity, n (%)	283 (92.8)
Clinical, HIV- and cART-related factors	
Main route for HIV acquisition, n (%)	
Homosexual/ Bisexual	262 (85.9)
Heterosexual	27 (8.9)
IVDU	4 (1.3)
Other or unknown	12 (3.9)
cART, n (%)	289 (94.8)
Time on cART, median years (IQR)	4.4 (1.9–9.3)
HIV RNA, n (%)	
Not detected	221 (72.5)
Detectable <40 copies/ml	47 (15.4)
> 40 copies/ml	37 (12.1)
CD4+ cell count, n (%)	
≥ 500/μl	243 (79.7)
201–499/μl	54 (17.7)
≤ 200/μl	8 (2.6)
Patient-reported health behaviours	
Cigarette smoking, n (%)	
Never	107 (35.1)
Former	53 (17.4)
Current	145 (47.5)
Use of recreational drug, n (%)	
Never	224 (73.4)
Former	34 (11.1)
Current	47 (15.4)
Current alcohol consumption (>3 units/daily), n (%)	11 (3.6)
Patient-reported sexual behaviours	
Number of lifetime sexual partners, median (IQR)	
Heterosexual males	10 (5.5–35)
Bi-sexual males	100 (30–300)
Homosexual males	100 (20–400)
Receptive oral sex ever, n (%)	275 (90.2)
Sexual activity in previous 12 months, n (%)	284 (93.1)
Type of sexual partner in previous 12 months, n (%)^a^	
Heterosexual	31 (10.9)
Bi-sexual	44 (15.5)
Homosexual	209 (73.6)
Number of partners in previous 12 months, median (IQR)	
Heterosexual	1 (1–2)
Bi-sexual	7 (3–19)
Homosexual	4 (1–10)
Previous or current anal condylomatosis, n (%)	112 (36.7)

cART: combination antiretroviral therapy; IVDU: intravenous drug use

^a^percentages calculated on 284 sexually active men (93.1% of the total) in the last 12 months

Health behaviours assessed in the interview showed that 64.9% of patients stated cigarette smoke-exposure (17.4% previously and 47.5% currently), 26.5% reported previous or current recreational drug use, and 3.6% declared current alcohol intake. Sexual history in the patient-reported questionnaire showed at that 152 men (49.8%) counted more than 100 sexual partners in lifetime. In the previous 12 months, 21 men (6.9%) declared no sexual activity, while 97 (31.8%) recalled 10 or more partners. The 284 patients who were sexually-active in the preceding 12 months, recalled the following type of sexual partners: only heterosexual in 31 (10.9%), both sexes in 44 (15.5%), and only homosexual in 209 (73.6%) cases. Median number of sexual partners in the previous 12 months were: 1 for heterosexuals (IQR: 1–2), 7 for bisexuals (IQR: 3.0–18.8), and 4 for homosexuals (IQR: 1–10). Overall, 275 (90.2%) patients reported receptive oral sex. None of the participating patients had been vaccinated for HPV.

### Oral HPV detection

HPV DNA was found in oral rinses of 64 out of 305 HIV-positive men (20.9%). HPV genotype was detected by MY09/11 primers in all but 12 cases, in which direct sequencing identified: HPV13 *n* = 2; HPV22 *n* = 3; HPV32 *n* = 8, HPV97 n = 2; HPV107 *n* = 1; HPV145 *n* = 1.

**Fig. 1:**
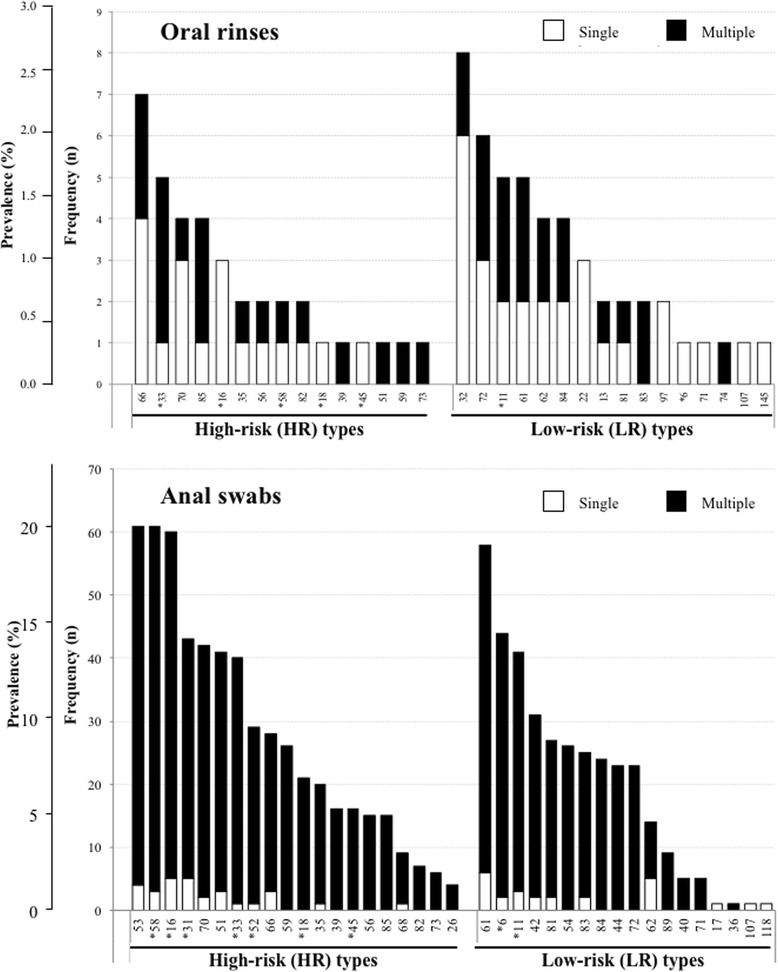
Distribution of HPV types found in the oral rinses of 64 HPV DNA-positive subjects (upper panel) or in anal swabs of 260 HPV DNA-positive subjects (lower panel), shown by multiplicity (single or multiple infection) and according to High-Risk (HR) or Low-Risk (LR) types. HPV types included in the nonavalent vaccine are indicated with an asterisk

The upper panel of Fig. 1 shows the different HPV types detected in the oral cavity, according to multiplicity of infection and risk types. Multiple HPV infections were detected in oral rinses in 18 cases (28.1%): two types in 16 patients, and three to four types in one case each. In HPV DNA-positive oral rinses, alpha-3 species were found in 23 (HPV61 *n* = 5, HPV62 *n* = 4, HPV72 *n* = 6, HPV81 and HPV83 in 2 cases each, HPV84 *n* = 4), alpha-7 in 14 (HPV18, HPV39, HPV45 and HPV59 in 1 case each, HPV70 and HPV85 in 4 cases each, HPV97 *n* = 2), and alpha-9 in 12 cases (HPV16 *n* = 3, HPV33 *n* = 5, HPV35 and HPV58 in 2 cases each). HPV16 and HPV18 were found in three and one oral samples, respectively. Beta-2 species were detected in 5 samples (HPV22 *n* = 3, HPV107 and HPV145 in 1 case each). HR types were found in 50% oral rinses with detectable HPV DNA (*n* = 32), and were more frequently observed in presence of multiple than of single HPV types (77.8% versus 39.1%; *p* = 0.005).

### Cytological examination of oral rinses

Oral rinses samples were not adequate for cytological examination in 27 patients because of low cellularity. Among 278 cases, cytology was normal in 228 (82.0%) cases, while cellular atypia (ASC-US or more) was found in 50 patients: ASC-US *n* = 49 (17.6%) and H-SIL *n* = 1. In Table 2, the distribution of cytological findings according to HPV risk group is shown. The risk of cellular atypia (ASC-US or more) was higher in presence of HR types when compared to subjects harbouring only LR types (OR 2.31; 95% CI 0.83–6.69; *p* = 0.050).

**Table 2:** Cytological findings in oral rinses of patients with oral HPV infection according to presence of HPV and oncogenic risk

	Total	Negative	HR	LR
		n (%)	n (%)	n (%)
Not adequate	27	26 (96.3)	1 (3.7)	–
Negative	228	191 (83.8)	15 (6.7)	22 (9.5)
ASC-US	49	24 (49.0)	15 (30.6)	10 (20.4)
H-SIL	1	–	1 (100)	–
Total	305	241	32	32

LR: HPV infection sustained by only low risk HPV types; HR: HPV infection sustained by at least one high risk HPV type; Negative: negative for intraepithelial lesion or malignancy; ASC-US: atypical squamous cells of undetermined significance; H-SIL: high grade squamous intraepithelial lesion

### *Co-occurrence* of HPV DNA at the oral cavity and at the anal site

HPV DNA was detected at the anal site in 260 out of 305 (85.2%) male patients. Thus, the proportion of subjects with positive HPV DNA at the oral cavity was significantly lower when compared to that of persons with HPV DNA at the anal site (21.0% versus 85.2%; *p* < 0.001). The finding of multiple HPV infection was more frequent in anal (206/260) than in oral (18/64) samples (79.2% versus 28.1%; *p* < 0.001). Out of 265 HPV DNA-positive patients regardless the anatomic site, only in 59 cases (22.3%) HPV DNA was recovered in oral rinses and in anal samples at the same time (19.3% of the overall cohort). Detection of HPV at the oral cavity tended to be more frequent in men with HPV DNA detected at anal site when compared to those with negative results (22.7% versus 11.1%, *p* = 0.078). Concerning specific HPV types, among the 59 patients with detectable HPV DNA at both anatomical sites, 51 (86.4%) had completely different HPV types. Out of the remaining eight, seven cases shared only one HPV type (HPV11 *n* = 2; HPV61, HPV81 and HPV83 in 1 case each among LR HPV types; HPV51 and HPV66 in 1 case each among HR HPV types), while one patient harboured the same double infection by HR HPV39 and HPV59 types at both sites. In Fig. 1, the distribution of HPV types detected at the anal site are shown by multiplicity and oncogenicity of HPV types.

### Variables predictive of positive oral HPV DNA

Table 3 shows the univariate analysis that found significantly higher odds for oral HPV DNA detection in men who have had more than 5 sexual partners in the previous 12 months (OR 2.72; 95% CI 1.39–5.34; *p* = 0.004) or more than 100 partners in lifetime (OR 5.77; 95% CI 1.31–25.35; *p* = 0.020). Further, a significant association between oral HPV DNA and self-reported history of anal/genital condylomatosis (OR 1.86; 95% CI 1.06–3.24; *p* = 0.030), use of recreational drugs (OR 2.0; 95% CI 1.01–3.98, *p* = 0.048), and current CD4 cell count below 200/μL when compared to higher values (OR 12.36; 95% CI 2.43–62.83; *p* = 0.002) was found.

**Table 3:** Factors associated with positive oral HPV DNA at univariable and multivariable analysis

	Positive HPV DNA (n/total)	Univariate analysis		Multivariable analysis^§^	
		OR (95% CI)	*p*	aOR (95% CI)	*p*
Age, years					
≥ 45	26/146	1		1	
< 45	38/159	1.45 (0.83–2.54)	0.193	1.09 (0.59–2.00)	0.791
Current use of recreational drugs					
No or former	49/258	1		1	0.422
Yes	15/47	2.0 (1.01–3.98)	0.048*	1.36 (0.64–2.90)	
Current cigarette smoking					
No or former	28/160	1			
Yes	36/145	1.58 (0.89–2.71)	0.118		
Duration of HIV infection, years					
< 2	11/55	1			
2–4	13/80	0.78 (0.32–1.89)	0.576		
> 4	35/142	1.23 (0.58–2.61)	0.587		
cART					
No	4/16	1			
Yes	60/289	0.79 (0.25–2.52)	0.686		
CD4+ cell count/μl					
≥ 200	58/297	1		1	
< 200	6/8	12.36 (2.43–62.83)	0.002*	12.58 (2.19–72.30)	0.005*
HIV RNA, copies/mL,					
Not detected or detectable ≤40	52/268	1	0.072^‡^	1	0.277
> 40	12/37	1.99 (0.94–4.23)		1.59 (0.69–3.70)	
Type of sexual partner					
Heterosexual	5/33	1			
Homosexual	28/135	1.47 (0.52–4.14)	0.471		
Bisexual	31/137	1.64 (0.58–4.60)	0.349		
N. of sexual partners in lifetime					
< 10	2/27	1			
10–100	14/126	1.56 (0.33–7.32)	0.571		
> 100	48/152	5.77 (1.31–25.35)	0.020*		
N. of sexual partners in the last 12 months					
0–1	16/120	1		1	
2–5	17/80	1.75 (0.83–3.72)	0.143	2.01 (0.90–4.49)	0.090^‡^
> 5	31/105	2.72 (1.39–5.34)	0.004*	2.78 (1.30–5.93)	0.008*
Oral sex					
No or insertive	3/30	1			
Receptive/both	61/275	2.57 (0.75–8.74)	0.132		
Anal HPV DNA					
Negative	5/45	1		1	
Positive	59/260	2.35 (0.89–6.22)	0.086^‡^	1.72 (0.63–4.68)	0.289
History of anal/genital condylomatosis					
No	33/193	1		1	
Yes	31/112	1.86 (1.06–3.24)	0.030*	1.42 (0.78–2.58)	0.252

*OR* odds ratio, *aOR* adjusted odds ratio, *cART* combination antiretroviral therapy

§ In the model all variables found to be associated with positive oral HPV DNA at univariate analysis at *p* < 0.10 were included, forcing age. The variable “N. of sexual partner in the previous 12 months” instead of “N. of sexual partners in lifetime” was used to avoid collinearity

* *p* < 0.05; ‡ *p* < 0.10

At multivariable analysis, after adjusting for age as well as all variables found to be associated with positive oral HPV DNA at univariate analysis (*p* < 0.01) and choosing to include “number of sexual partner in the previous 12 months” instead of “number of lifetime partners” to avoid collinearity, a significant independent association between detectable oral HPV DNA and the following conditions was confirmed: CD4 cell count ≤200/μL versus > 200/μL (OR 12.58; 95% CI 2.19–72.30; *p* = 0.005) and to report >5 sexual partners in the previous 12 months versus 0–1 partner (OR 2.78; 95% CI 1.30–5.93; *p* = 0.008).

### Vaccine-targeted HPV types

A minimum of one nonavalent vaccine-targeted HPV type was found in 17 out of 64 patients (26.6%) with positive oral HPV DNA, and in 199 out of 260 of patients (76.5%) with HPV positivity at the anal site. The nonavalent vaccine-targeted vaccine would have covered all identified HPV types in 11/17 (64.7%) of patients with HPV at the oral cavity and in 33/199 (16.6%) of patients with HPV at the anal site (Fig. 1).

## Discussion

In this clinic-based study on Italian HIV-positive men (predominantly MSM) observed within an anal cancer screening, HPV was detected in 20.9% of subjects at the oral cavity with a significantly lower prevalence than that observed at the anal site. Severe immunodepression and number of sexual partners in the previous 12 months, but not anal HPV detection, were significantly associated with a higher odd of positive oral HPV DNA. Co-occurrence of HPV DNA at the oral cavity and at the anal site was not found, and with few exceptions in patients with infection at both sites HPV types were completely different.

In our study, positive HPV DNA in oral rinses was found in approximately one out of five tested HIV-infected patients, confirming that HPV detection at the oral cavity is quite common in HIV-positive men, especially if MSM. This is in accordance with previous studies [10] and with data from a recently published meta-analysis, that calculated a pooled oral HPV prevalence in MSM with HIV infection of 28.9% (95% CI 19.1–38.7) [12]. Further, our results confirm that the prevalence of positive oral HPV DNA in the HIV-infected population in Italy is considerably higher than in young Italian HIV-uninfected adults, where it has been shown around 4% [29]. Overall, these findings underscore the importance to investigate the oral cavity of HIV-positive men, particularly if MSM, for the presence of HPV to identify patients eventually at higher risk of OSCC development and who might need to undergo cytological examination of oral rinses and stomatological evaluation during follow-up.

With regards to factors predictive of positive oral HPV DNA, our finding of CD4 cell count below 200/μL and higher number of sexual partners in the previous 12 months, agrees with some previous studies [3, 12]. In addition, Beachler et al. [12] reported an increased incidence of oral HPV infection among HIV-infected subjects with decreased CD4 cell count and in those reporting high numbers of partners, whereas male sex, older age and current smoking increased the risk of oral HPV persistence.

In our study, detection of HPV DNA at the oral cavity and at the anal site was not associated to each other. Furthermore, in patients with positive HPV DNA at both anatomical sites, HPV strains were mostly different. The absence of concordance has also been observed in another few studies conducted in HIV-positive men [7, 11, 14, 30, 31]. It is possible that the considerably lower prevalence of HPV DNA at the oral cavity when compared to the anus, together with the lack of concordance in HPV type-specific infections, allows the hypothesis that HPV at the oral site is acquired independently of ano-genital infection or perhaps at different points in time. In fact, self-inoculation by the patient from the ano-genital tract to the oral cavity as transmission route [32], seems unlikely even if considering clearance rates that could differ between the anatomical regions. Nevertheless, one could speculate that, because of differences in local mucosal immunity, non-genital site infections are less likely to reactivate from latency when compared to genital infections, where micro-traumatisms and occurrence of sexually transmitted diseases may decrease the ability to clear ano-genital HPV types in respect to oral infections.

In our study, prevalence of most frequent oral HPV types was HPV66 and HPV33 among HR, and HPV32, HPV72, HPV11 and HPV61 among LR. HPV16 was found in only 1.0% of cases. This finding differed from previous studies, that showed HPV16 as the predominant type [6, 33], and may have epidemiological implications. In fact, HPV16 is found in more than 85% of HPV OSSC [6], and a case-control study estimated that oral HPV16 confers a 50-fold increase in the odds of HPV-driven oro-pharyngeal cancer [31].

In our study population, if the patient had been vaccinated before exposure, the nonavalent vaccine would have targeted at least one HPV strain in only about 27% patient with detectable HPV DNA at the oral cavity and in 77% at the anal site. Notably though, nonavalent vaccine would have covered all identified HPV types in more than 60% of patients with oral HPV DNA, but only less than 20% of patients with HPV DNA at the anal site. Based on these findings, it may be reasonable to investigate the efficacy of nonavalent vaccines in preventing oral HPV infections.

Our study has some limitations that should be mentioned. Since the tested men have been send at anal cancer screening program by the caring HIV physician, selection bias may have occurred by choosing patients at higher risk of sexually transmitted diseases in general and HPV specifically. Moreover, since a non-standardized interviewer-administered questionnaire was used to collect information regarding sexual history and practices, interview bias may have occurred with distortion of responses by the interviewer or elusion and lies in the answers of the patients. However, our interview was mostly build on fixed-wording questions and multiple-choice answers, reducing the risk of bias.

## Conclusions

In this study on Italian HIV-positive men (predominantly MSM), oral HPV DNA was detected in approximately one fifth of tested men, but prevalence was significantly lower than that observed at the anal site. Low CD4 cell count and increasing number of recent sexual partners significantly increased the odds of positive oral HPV detection. The absence of co-occurrence of HPV DNA at the oral cavity and at the anal site may suggest different routes or timing of infection.

## Abbreviations

ASC-US: Atypical squamous cells of undetermined significance
cART: combination antiretroviral therapy
HPV: Human Papilloma Virus
HR: Hgh Risk HPV types
H-SIL: High grade squamous intraepithelial lesion
LR: Low risk HPV types
L-SIL: Low grade squamous intraepithelial lesion
NILM: Negative for intraepithelial lesion or malignancy
OSCC: Oro-pharyngeal squamous cell carcinoma
